# HLA-B27 as a predictor of effectiveness of treatment with TNF inhibitors in axial spondyloarthritis: data from the Swiss Clinical Quality Management Registry

**DOI:** 10.1007/s10067-022-06490-8

**Published:** 2022-12-27

**Authors:** Fabienne Fröhlich, Raphael Micheroli, Monika Hebeisen, Seraphina Kissling, Kristina Bürki, Pascale Exer, René Bräm, Karin Niedermann, Burkhard Möller, Michael J. Nissen, Diego Kyburz, Michael Andor, Oliver Distler, Almut Scherer, Adrian Ciurea

**Affiliations:** 1 Department of Rheumatology, Zurich University Hospital, University of Zurich, Gloriastrasse 25, CH-8091 Zurich, Switzerland; 2 Department of Dermatology, Zurich University Hospital, Zurich, Switzerland; 3 Swiss Clinical Quality Management Foundation, Zurich, Switzerland; 4 Gemeinschaftspraxis Rheuma-Basel, Basel, Switzerland; 5 Swiss Ankylosing Spondylitis Association, Zurich, Switzerland; 6 School of Health Sciences, Institute of Physiotherapy, Zurich University of Applied Sciences, Winterthur, Switzerland; 7 Department of Rheumatology and Immunology, Inselspital, Bern, Switzerland; 8 Department of Rheumatology, Geneva University Hospital, Geneva, Switzerland; 9 Department of Rheumatology, University Hospital Basel, University of Basel, Basel, Switzerland; 10 Rheumatologie im Zürcher Oberland, Uster, Switzerland

**Keywords:** Axial spondyloarthritis, HLA-B27, TNF inhibition, Treatment response

## Abstract

**Objective:**

To explore the impact of the human leucocyte antigen (HLA)-B27 on the effectiveness of tumor necrosis factor inhibitors (TNFi) in patients with axial spondyloarthritis (axSpA).

**Methods:**

A total of 1109 patients with available HLA-B27 status (831 B27+ patients and 278 B27− patients) fulfilling the Assessment of Spondyloarthritis international Society classification criteria for axSpA from the prospective Swiss Clinical Quality Management Registry initiating a first TNFi were included. Drug retention was investigated with multiple adjusted Cox proportional hazard models with imputation of missing values. Multiple-adjusted logistic regression analyses were used to assess the proportion of patients reaching 50% reduction in the Bath Ankylosing Spondylitis Disease Activity Index (BASDAI50) at 1 year.

**Results:**

B27+ and B27− patients differed with regard to age, sex, BASDAI, C-reactive protein (CRP), body mass index, enthesitis, uveitis, and classification status. After adjustment for potential confounders for the relationship between HLA-B27 and drug effectiveness (sex and family history of spondyloarthritis), a higher risk of drug discontinuation was found in B27− patients (HR 1.53, 95% CI 1.27–1.83). This difference decreased after additional adjustment for parameters which may act as mediators (HR 1.30, 95% CI 1.30–1.55). Male sex and elevated C-reactive protein (CRP) levels were consistently associated with longer retention. Comparable results were obtained for BASDAI50 responses.

**Conclusion:**

The HLA-B27 genotype is an important predictor of treatment effectiveness. Male sex and CRP seem, however, to better describe variability of response in individual patients. This data may help avoiding potential discrimination of B27− individuals with regard to TNFi initiation.

**Table:** 

**Key Points** • *HLA-B27 is a predictor of effectiveness of TNF inhibitors in axial spondyloarthritis.* • *Variability of response in individual patients is better defined by sex and objective markers of disease activity, such as C-reactive protein.*

## Introduction

The pathogenic mechanisms underlying the strong association between the human leucocyte antigen (HLA)-B27 and ankylosing spondylitis (AS)—discovered 50 years ago [1, 2]—remain incompletely understood. Several mutually not exclusive hypotheses have been put forward for its explanation [3, 4]. Data on phenotypic differences between HLA-B27 positive (B27+) and HLA-B27 negative (B27−) individuals with axial spondyloarthritis (axSpA) continue to accrue and have recently been reviewed [5, 6]. HLA-B27 has been identified as a predictor of good response to biologic disease-modifying antirheumatic drugs (bDMARDs) and especially tumor necrosis factor inhibitors (TNFi) in some studies [7–9], but not in others [10–13]. Moreover, it remains unclear whether differences in treatment response are due to the pathogenic mechanisms of the molecule in itself or mediated through clinical, laboratory, or imaging features that delineate the specific phenotype. The aim of this study is to explore the influence of HLA-B27 on the effectiveness of treatment after start of a first TNFi in individuals with axSpA.

## Methods

### Study population

This study is a longitudinal analysis of the ongoing Swiss Clinical Quality Management (SCQM) registry of patients with a clinical diagnosis of axSpA [14] recruited from January 2005 to January 2021. Assessments at inclusion and annual visits were performed according to the recommendations of ASAS [15]. bDMARD-naïve patients were included in the current study if they fulfilled the ASAS criteria for axial spondyloarthritis [16], started treatment with a first TNFi after inclusion into the registry, and if baseline disease activity information at initiation of a first TNFi were available. The study was approved by the Ethics Commission of the Canton of Zurich (KEK-ZH-Nr. 2014-0439). Written informed consent was obtained from all patients.

### Effectiveness of anti-TNF treatment

Drug retention was considered the primary outcome using start and stop dates indicated by the treating rheumatologist. Observations were censored at the last visit recorded in the SCQM database. Treatment response—defined as the proportion of patients reaching a 50% reduction in the Bath Ankylosing Spondylitis Disease Activity Index (BASDAI50)—was assessed in an exploratory analysis in patients with available disease activity measurements at 1 year (+/− 6 months), independently on whether treatment was stopped or changed (intention-to-treat analysis). The large window of response assessment was mandated by the structure of SCQM as annual follow-up visits recommended after inclusion did not necessarily match yearly intervals after initiation of treatment.

### Statistical analysis

We compared baseline characteristics between groups using the Fisher’s exact test for categorical variables and the Mann-Whitney test for continuous variables. The tests were two-sided, with a significance level set at 0.05. Drug retention was described with Kaplan-Meier plots. We utilized the Log-rank test to test for differences between retention in B27+ and B27− individuals and a multiple adjusted Cox proportional hazard model to estimate a covariate-adjusted effect of HLA-B27 status on drug retention. In a simplified model (referred to as Model 1 in the tables of the “Results” section) the analysis was only adjusted for sex and family history of SpA, as both could potentially be regarded as confounders (variables potentially affecting both exposure of interest and the outcome). Other baseline factors that might be influenced by the HLA-B27 genotype are lying in the causal path to treatment survival and are therefore regarded as potential mediators, not confounders, for our analysis. We therefore included the following parameters in a second adjusted analysis: age, sex, family history of spondyloarthritis (SpA), elevated CRP status, BASDAI, presence of enthesitis, ever uveitis, education, current smoking, body mass index (BMI), response to non-steroidal anti-inflammatory drugs (NSAIDs), and classification status as nonradiographic vs. radiographic axSpA (referred to as model 2 in the tables of the “Results” section). We tested for the presence of an interaction between HLA-B27 status and sex. The significance of the unadjusted difference in BASDAI50 responses at 1 year was assessed using the Fisher’s exact test. Logistic regression analysis was used to estimate an adjusted ratio for BASDAI50, with adjustment for the same parameters as in the Cox regression analysis.

Multiple imputation by chained equations was used to deal with missing baseline covariates. Imputation was performed separately for the retention and the BASDAI50 response analyses. A total of 30 imputation data sets from 50 iterations were used for both imputations. Out of 1009 patients with known HLA-B27 status in the retention analysis, 672 (66.6%) had at least one missing value in one of the 13 variables used in model 2. The proportion of missing values per variable varied from 0 to 38%. With regard to the BASDAI50 response analysis, 313 out of 581 patients (57%) had at least one missing value. The proportion of missing values per covariate varied from 0 to 29%. Predictive mean matching was used to impute continuous variables, logistic regression for binary variables and polynomial regression for parameters with more than two levels. Variables used in the imputation models included all variables used in models 1 and 2, and additional variables not included in the models that inform on disease activity, function, and quality of life. The Nelson-Aalen estimator was added to the variable space as a measure for the cumulative hazard in the retention data set. For the BASDAI50 response data set, the BASDAI score at follow-up was added to the variables space. BMI category was passively imputed from weight and height. Convergence of imputations was assessed by visual inspection of the mean and variance changes by iteration and dataset. Pooling of model estimates was performed according to Rubin’s rule. The MICE package version 2.30 was used for the imputation. The R statistical software was used for all analyses.

## Results

### Baseline characteristics

**Fig. 1:**
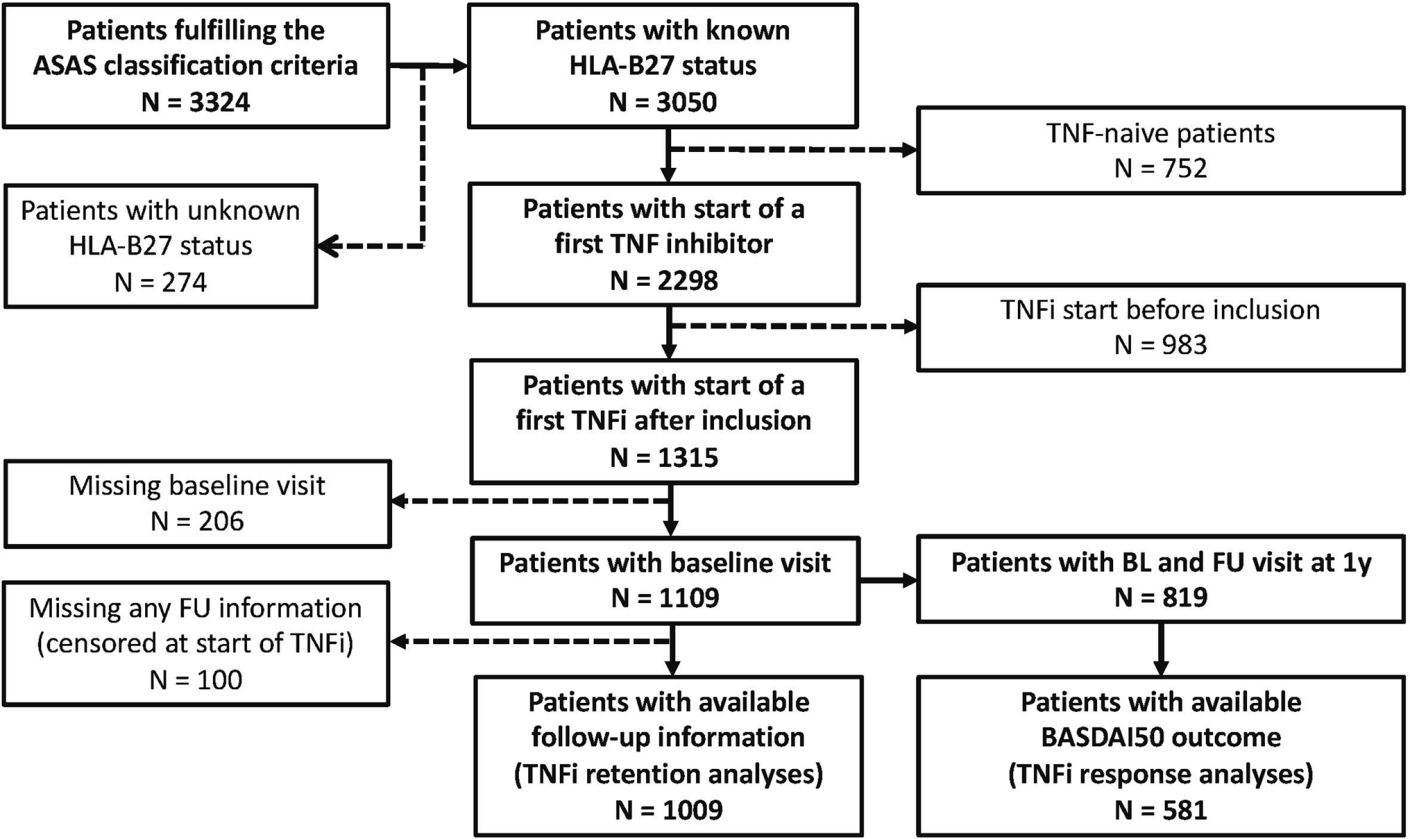
Disposition of patients with axial spondyloarthritis in the SCQM registry. ASAS = Assessment in SpondyloArthritis international Society; BL = baseline; FU = follow-up; HLA-B27 = human leucocyte antigen B27; TNFi = tumor necrosis factor inhibitor; y = year

Disposition of patients fulfilling the ASAS classification criteria in the SCQM cohort is depicted in Fig. 1. The HLA-B27 status was known in 3050 out of 3324 axSpA patients (91.8%). A total of 1315 patients with available HLA-B27 status started a first TNFi after inclusion in SCQM and 1109 patients had an available visit at baseline. Baseline characteristics of these patients at treatment start are shown in Table 1. After exclusion of 100 patients lacking any follow-up information and censored at baseline, 1009 patients were available for treatment retention analyses (607 patients with known TNFi stop date and 402 patients censored at last visit in SCQM). The characteristics of patients included in the TNFi retention analyses are also shown in Table 1. B27+ patients had an earlier onset of disease and a longer disease duration and were in a higher proportion of male sex. While the proportion of patients with elevated CRP was higher in B27+ patients, patient-reported disease activity as assessed by the BASDAI was slightly higher in B27− patients. No significant differences between the two groups were found with respect to impairments in function, mobility, and health-related quality of life. Peripheral arthritis and dactylitis were evenly distributed between the two groups. While hip arthritis was more prominent in B27+ patients, the frequency of enthesitis was higher in B27− patients. With regard to extra-musculoskeletal manifestations, B27+ patients were more frequently affected by uveitis, while the prevalence of psoriasis and of inflammatory bowel disease (IBD) was slightly higher in B27− patients. B27+ patients had a lower BMI and more often higher education levels than B27− patients.

**Table 1:** Baseline characteristics of axSpA patients starting a first TNFi

Parameter	A. All patients starting a first TNFi				B. Patients in drug retention analysis			
	*N* 1109	B27 pos *N* **= **831	B27 neg *N =* 278	*P*	*N* 1009	B27 pos *N =* 762	B27 neg *N =* 247	*P*
Male sex, *N* (%)	1109	527 (63.4)	109 (39.2)	<0.001	1009	490 (64.3)	96 (38.9)	<0.001
Age, years	1109	38.5 (11.8)	40.4 (10.0)	<0.001	1009	38.4 (11.7)	40.8 (9.8)	<0.001
Age at onset, years	1099	25.5 (7.9)	30.9 (8.9)	<0.001	1000	25.3 (7.7)	30.9 (8.9)	<0.001
Symptom duration, years	1099	13.0 (11.2)	9.5 (9.2)	<0.001	1000	13.1 (11.0)	9.8 (9.3)	<0.001
Diagnostic delay, years	1094	5.8 (7.6)	6.0 (7.8)	0.48	996	6.0 (7.7)	6.3 (8.0)	0.29
Family history SpA, *N* (%)	985	278 (37.9)	59 (23.5)	<0.001	902	252 (37.3)	55 (24.3)	<0.001
Radiographic axSpA, *N* (%)	673	384 (76.0)	101 (60.1)	<0.001	626	359 (75.7)	91 (59.9)	<0.001
BASDAI	897	5.4 (2.0)	5.8 (2.0)	0.006	821	5.4 (2.0)	5.8 (2.0)	0.01
ASDAS	831	3.4 (1.0)	3.3 (0.8)	0.21	759	3.4 (0.9)	3.3 (0.8)	0.26
CRP (mg/l), median (IQR)	1024	14.8 (20.4)	9.2 (13.1)	<0.001	932	15.1 (20.8)	9.5 (13.6)	<0.001
Elevated CRP, *N* (%)	1022	415 (54.2)	93 (36.3)	<0.001	930	383 (54.5)	84 (37.0)	<0.001
BASFI	898	3.8 (2.5)	4.1 (2.4)	0.07	820	3.8 (2.5)	4.1 (2.4)	0.06
BASMI	924	2.1 (2.0)	1.9 (1.6)	0.59	834	2.1 (2.0)	1.9 (1.5)	0.87
EQ-5D	878	57.5 (21.5)	54.8 (21.0)	0.05	801	57.5 (21.5)	54.7 (21.3)	0.07
Current arthritis, *N* (%)	1069	269 (33.7)	91 (33.7)	1.00	969	244 (33.4)	79 (33.0)	0.94
Current hip arthritis, *N* (%)	984	87 (11.9)	26 (10.3)	0.57	884	82 (12.4)	25 (11.3)	0.72
Number of swollen joints	1042	0.7 (2.1)	0.8 (2.2)	0.76	945	0.6 (1.8)	0.8 (2.2)	0.72
Current enthesitis, *N* (%)	1061	539 (68.0)	210 (78.4)	0.001	963	494 (68.1)	188 (79.0)	0.001
Modified MASES	1053	2.5 (3.0)	3.3 (3.3)	<0.001	955	2.4 (2.9)	3.4 (3.4)	<0.001
Dactylitis ever, *N* (%)	1101	90 (10.9)	31 (11.2)	0.91	1001	79 (10.5)	28 (12.3)	0.72
Uveitis ever, *N* (%)	1011	176 (23.3)	19 (7.4)	<0.001	930	162 (23.1)	19 (8.3)	<0.001
Psoriasis ever, *N* (%)	922	67 (9.8)	33 (13.9)	0.09	857	63 (9.8)	33 (15.2)	0.03
IBD ever, *N* (%)	1001	61 (8.2)	32 (12.6)	0.05	922	57 (8.2)	28 (12.3)	0.09
Current smoking, *N* (%)	857	244 (37.4)	72 (35.1)	0.56	781	224 (37.6)	66 (35.7)	0.66
Body mass index	1058	25.2 (4.5)	26.4 (5.0)	0.002				
Ever csDMARD, *N* (%)	1109	246 (29.6)	96 (34.5)	0.13	1009	226 (29.7)	94 (38.1)	0.01
Good response to NSAIDs, N(%)	923	566 (83.7)	175 (70.8)	<0.001	851	534 (84.8)	157 (71.0)	<0.001
Education	952			0.003	665			<0.001
Compulsory		102 (14.2)	55 (23.8)			89 (13.4)	52 (24.9)	
Vocational		404 (56.0)	128 (55.4)			377 (56.7)	110 (52.6)	
Academic		215 (29.8)	48 (20.8)			199 (29.9)	47 (22.5)	

**A**—All patients starting a first TNFi after inclusion in SCQM. **B**—Patients in the drug retention analysis. *ASDAS*, Ankylosing Spondylitis Disease Activity Score; *BASDAI*, Bath Ankylosing Spondylitis Disease Activity Index; *BASFI*, Bath Ankylosing Spondylitis Functional Index; *BASMI*, Bath Ankylosing Spondylitis Metrology Index; C-reactive protein (CRP) levels; *EQ-5D*, EuroQol 5-domains; *HLA-B27*, human leucocyte antigen B27; *IBD*, inflammatory bowel disease; *MASES*, Maastricht Ankylosing Spondylitis Enthesitis Score; modification refers to the inclusion of the plantar fascia in the count. *NSAIDs*, nonsteroidal anti-inflammatory drugs; *TNFi*, tumor necrosis factor inhibitor

### TNFi retention

Median TNFi retention was longer in B27+ versus B27− patients: 3.68 years (95% confidence interval (CI) 3.10–4.243) versus 1.33 years (95% CI 0.96–2.09), respectively (*P*<0.001). With regard to sex, median retention was 4.59 years (3.63–5.89) in B27+ male patients versus 2.81 years (1.87–3.63) in B27+ female patients, and 2.18 years (1.49–5.26) versus 0.99 years (0.61–1.43) in B27− male versus female patients, respectively, *P*-value <0.001.

The hazard of discontinuing TNFi was higher in B27− patients after adjustment for sex and positive family history for SpA in a Cox proportional hazard model (hazard ratio (HR) 1.53, 95% CI 1.27–1.83; model 1 in Table 2). In this model, female sex was also associated with an increased hazard of discontinuing TNFi (HR 1.57, 95% CI 1.33–1.85). The results were confirmed in a complete case analysis (model 1 in Table 3). After adjustment for additional parameters found to differ between B27+ and B27− individuals, the hazard of stopping treatment with TNFi in B27− patients decreased (HR 1.30, 95% CI 1.07–1.58 after multiple imputation of missing covariate data and HR 1.10, 95% CI 0.78–1.55 in a complete case analysis; Tables 2 and 3, respectively). Elevated CRP status was associated with better TNFi retention in both analyses. We found no interaction between the HLA-B27 status and sex. Moreover, classification status as nr-axSpA versus r-axSpA was not identified as an important independent predictor of drug retention in these models.

**Table 2:** Multiple adjusted Cox proportional hazard model for analysis of drug discontinuation of a first TNF inhibitor in HLA-B27 negative vs. positive patients after multiple imputation of missing covariate data

Analysis based on multiple imputation of missing data	Model 1			Model 2		
Variable	HR	95% CI	*P*	HR	95% CI	*P*
HLA-B27 negative (Ref: HLA-B27 positive)	**1.53**	**1.27; 1.83**	**<0.001**	**1.30**	**1.07; 1.58**	**0.01**
Female sex (Ref: male sex)	**1.57**	**1.33; 1.85**	**<0.001**	**1.53**	**1.28; 1.83**	**<0.001**
Family history SpA positive	0.99	0.83; 1.18	0.87	1.01	0.84; 1.21	0.92
Elevated CRP				**0.67**	**0.56; 0.79**	**<0.001**
BASDAI				1.00	0.95; 1.05	0.94
Enthesitis				1.00	0.82; 1.22	0.99
Education vocational (Ref: compulsory)				0.88	0.69; 1.12	0.31
Education academic (Ref: compulsory)				1.03	0.79; 1.35	0.81
BMI 25-30 (Ref: BMI <25)				1.14	0.94; 1.38	0.19
BMI >30 (Ref: BMI ≤25)				**1.30**	**1.02; 1.65**	**0.03**
Age				1.00	0.99; 1.01	0.52
Uveitis ever				**0.76**	**0.61; 0.94**	**0.01**
Good response to NSAIDs				0.90	0.72; 1.12	0.33
nr-axSpA (Ref: r-axSpA)				1.20	0.98; 1.47	0.08
Current smoking				1.10	0.90; 1.34	0.34

Statistically significant results are shown in bold. *BASDAI*, Bath Ankylosing Spondylitis Disease Activity Index; *BASMI*, Bath Ankylosing Spondylitis Mobility Index; *BMI*, body mass index; *CRP*, C-reactive protein; *HLA-B27*, human leucocyte antigen-B27; *nr-axSpA*, nonradiographic axial spondyloarthritis; *NSAIDs*, non-steroidal anti-rheumatic drugs; *r-axSpA*, radiographic axial spondyloarthritis; *Ref*, reference; *TNF*, tumor necrosis factor

**Table 3:** Multiple adjusted Cox proportional hazard model for analysis of drug discontinuation of a first TNF inhibitor in HLA-B27 negative vs. positive patients with complete availability of data on covariates

Complete case analysis	Model 1			Model 2		
Variable	HR	95% CI	*P*	HR	95% CI	*P*
HLA-B27 negative (Ref: HLA-B27 positive)	**1.58**	**1.30; 1.91**	**<0.001**	1.10	0.78; 1.55	0.58
Female sex (Ref: male sex)	**1.50**	**1.26; 1.78**	**<0.001**	**1.65**	**1.20; 2.26**	**0.002**
Family history SpA positive	1.00	0.84; 1.20	0.97	1.09	0.81; 1.46	0.56
Elevated CRP				**0.71**	**0.53; 0.95**	**0.02**
BASDAI				1.00	0.93; 1.08	0.91
Enthesitis				0.77	0.56; 1.07	0.12
Education vocational (Ref: compulsory)				0.80	0.51; 1.26	0.34
Education academic (Ref: compulsory)				0.91	0.56; 1.49	0.72
BMI 25-30 (Ref: BMI <25)				1.15	0.83; 1.60	0.40
BMI >30 (Ref: BMI ≤25)				1.40	0.90; 2.19	0.13
Age				1.00	0.98; 1.01	0.74
Uveitis ever				0.91	0.65; 1.29	0.60
Good response to NSAIDs				1.17	0.80; 1.70	0.41
nr-axSpA (Ref: r-axSpA)				1.09	0.78; 1.53	0.61
Current smoking				1.08	0.80; 1.46	0.62

Analysis performed in 902 patients (545 events) in model 1 and 337 patients (208 events) in model 2. Statistically significant results are shown in bold. *BASDAI*, Bath Ankylosing Spondylitis Disease Activity Index; *BASMI*, Bath Ankylosing Spondylitis Mobility Index; *BMI*, body mass index; *CRP*, C-reactive protein; *HLA*-*B27*, human leucocyte antigen-B27; *nr-axSpA*, nonradiographic axial spondyloarthritis; *NSAIDs*, non-steroidal anti-rheumatic drugs; *r-axSpA*, radiographic axial spondyloarthritis; *Ref*, reference; *TNF*, tumor necrosis factor

### Treatment response

Out of 891 patients with available baseline and follow-up visits, BASDAI at 1 year was available in 581 patients (70.8%) (Fig. 1). BASDAI50 response was analysed in this population. It was achieved by 32% of B27− versus 50% of B27+ patients (OR 0.48, 95% CI 0.30 to 0.74, *P*<0.001, Table 4). Adjustment for sex and for a positive family history for SpA had only a minor influence on effect size: OR 0.57, 95% CI 0.37–0.89 in B27− versus B27+ patients (adjusted model 1 in Table 4). The difference in BASDAI50 response between B27− and B27+ patients further decreased after adjustment for a multitude of parameters defining the B27+ phenotype: OR 0.79, 95% 0.49–1.28 in B27− versus B27+ patients (adjusted model 2 in Table 4), corroborating the results found in the drug retention analyses. Our BASDAI50 response analyses performed after multiple imputation of missing covariate data were confirmed in complete case analyses (Table 5).

**Table 4:** BASDAI50 response in HLA-B27 negative versus HLA-B27 positive axSpA patients upon 1 year of treatment with a first TNF inhibitor after multiple imputation of missing covariate data

Analysis based on multiple imputation of missing covariate data	Unadjusted analysis					Adjusted model 1			Adjusted model 2		
Variables	B27−	B27+	OR	95% CI	*P*	OR	95% CI	*P*	OR	95% CI	*P*
HLA-B27 negative (Ref: HLA-B27 positive)	**32%**	**50%**	**0.48**	**0.30; 0.74**	**<0.001**	**0.57**	**0.37; 0.89**	**0.01**	0.79	0.49; 1.28	0.34
Female sex (Ref: male sex)						**0.56**	**0.39; 0.79**	**0.001**	**0.51**	**0.34; 0.76**	**0.001**
Family history SpA positive						**1.58**	**1.10; 2.27**	**0.01**	**1.59**	**1.07; 2.35**	**0.02**
Elevated CRP									**2.34**	**1.60; 3.42**	**<0.001**
BASDAI									1.09	0.99; 1.20	0.09
Enthesitis									1.35	0.88; 2.05	0.16
Education vocational (Ref: compulsory)									**2.49**	**1.38; 4.47**	**0.002**
Education academic (Ref: compulsory)									**2.03**	**1.07; 3.82**	**0.03**
BMI 25-30 (Ref: BMI <25)									0.76	0.50; 1.17	0.21
BMI >30 (Ref: BMI ≤25)									**0.41**	**0.23; 0.74**	**0.003**
Age									**0.97**	**0.95; 0.98**	**<0.001**
Uveitis ever									1.03	0.65; 1.64	0.89
Good response to NSAIDs									1.15	0.66; 1.99	0.62
nr-axSpA (Ref: r-axSpA)									0.64	0.40; 1.03	0.07
Current smoking									0.69	0.46; 1.02	0.06

Statistically significant results are shown in bold. *BASDAI*, Bath Ankylosing Spondylitis Disease Activity Index; *BASMI*, Bath Ankylosing Spondylitis Mobility Index; *BMI*, body mass index; *CRP*, C-reactive protein; *HLA-B27*, human leucocyte antigen-B27; *nr-axSpA*, nonradiographic axial spondyloarthritis; *NSAIDs*, non-steroidal anti-rheumatic drugs; *r*-*axSpA*, radiographic axial spondyloarthritis; *Ref*, reference; *TNFi*, tumor necrosis factor inhibitor

**Table 5:** BASDAI50 response in HLA-B27 negative versus HLA-B27 positive axSpA patients upon 1 year of treatment with a first TNF inhibitor in patients with complete availability of data on covariates

Complete case analysis	Adjusted model 1			Adjusted model 2		
Variables	OR	95% CI	*P*	OR	95% CI	*P*
HLA-B27 negative (Ref: HLA-B27 positive)	**0.56**	**0.35; 0.88**	**0.01**	0.80	0.39; 1.65	0.55
Female sex (Ref: male sex)	**0.57**	**0.40; 0.83**	**0.003**	**0.20**	**0.10; 0.40**	**<0.001**
Family history SpA positive	**1.56**	**1.08; 2.25**	**0.02**	**1.86**	**1.02; 3.43**	**0.04**
Elevated CRP				1.51	0.84; 2.72	0.17
BASDAI				**1.21**	**1.04; 1.42**	**0.02**
Enthesitis				**2.57**	**1.32; 5.12**	**0.006**
Education vocational (Ref: compulsory)				**3.22**	**1.14; 9.85**	**0.03**
Education academic (Ref: compulsory)				2.19	0.74; 6.92	0.17
BMI 25-30 (Ref: BMI <25)				0.67	0.34; 1.31	0.25
BMI >30 (Ref: BMI ≤25)				0.61	0.23; 1.55	0.30
Age				**0.97**	**0.94; 1.00**	**0.03**
Uveitis ever				**1.63**	**0.83; 3.25**	**0.16**
Good response to NSAIDs				1.00	0.45; 2.21	0.99
nr-axSpA (Ref: r-axSpA)				0.70	0.34; 1.65	0.55
Current smoking				0.86	0.47; 1.57	0.62

In the complete case analysis, 519 patients (819 observations) were included in model 1 and 250 patients (819 observations) in model 2. Statistically significant results are shown in bold. *BASDAI*, Bath Ankylosing Spondylitis Disease Activity Index; *BASMI*, Bath Ankylosing Spondylitis Mobility Index; *BMI*, body mass index; *CRP*, C-reactive protein; *HLA-B27*, human leucocyte antigen-B27; *nr-axSpA*, nonradiographic axial spondyloarthritis; *NSAIDs*, non-steroidal anti-rheumatic drugs; *r-axSpA*, radiographic axial spondyloarthritis; *Ref*, reference; *TNF*, tumor necrosis factor

## Discussion

Our data confirm that HLA-B27 represents an important predictor of response to treatment with TNFi in axSpA [7–9]. The effect size of HLA-B27 status on treatment effectiveness decreased after adjustment for baseline differences in parameters known to potentially influence the outcome. This finding suggests that the influence of the HLA-B27 genotype is mediated in part through parameters defining the B27+ phenotype, such as a higher load of inflammation. Indeed, the proportion of patients with elevated CRP, as well as the height of the CRP elevation were associated with HLA-B27 positivity. In contrast, male sex was independently associated with better TNFi effectiveness. In line with these results, HLA-B27 and male sex independently determined the likelihood of a positive MRI of the sacroiliac joints in patients with early inflammatory back pain [17].

HLA-B27 has been included in an early matrix and algorithm-based model to improve patient selection for treatment with TNFi [7]. It was combined with age, CRP level, functional status as assessed by the Bath Ankylosing Spondylitis Functional Index (BASFI) and the presence of enthesitis. As all these additional parameters seem differently expressed in the B27+ vs. B27− population, B27− patients might be discriminated a priori, if both genotype and phenotype-related parameters are considered. In a more recent attempt to predict probability of response to TNFi for individual patients using machine learning algorithms, the HLA-B27 genotype ranked relatively low for predicting major response if specific baseline characteristics, including CRP levels, were taken into account [18].

Classification as nonradiographic vs. radiographic axSpA had no significant impact on TNFi retention or response in our analysis, confirming data from another cohort [19]. Given the importance of HLA-B27 positivity for the fulfillment of the clinical arm of the ASAS classification criteria [16], this finding seems at least reassuring.

Inclusion of a large cohort of patients treated in real-life conditions, as well as the possibility to adjust for a multitude of factors known to affect treatment response, including smoking and obesity, represent strenghts of our analysis. Data on the presence of MRI inflammation prior to start of TNFi is, however, not available in SCQM. Drug retention was considered the primary outcome, as treatment response could only be assessed in patients with available outcome assessment in the respective timeframe.

In conclusion, while the HLA-B27 genotype is an important predictor of effectiveness of TNFi, sex and characteristics defined by the HLA-B27 phenotype—particularly markers of disease activity and systemic inflammation—seem to better describe variability of response in individual patients.
